# Design and Validation of a Chatbot-Based Cervical Cancer Screening Decision Aid for Women Experiencing Socioeconomic Disadvantage: User-Centered Approach Study

**DOI:** 10.2196/70251

**Published:** 2025-07-24

**Authors:** Alice Le Bonniec, Catherine Sauvaget, Eric Lucas, Abdelhak Nassiri, Farida Selmouni

**Affiliations:** 1 National Screening Service Dublin Ireland; 2 International Agency for Research on Cancer Lyon France; 3 University of Western Brittany Brest France

**Keywords:** cervical cancer screening, human papillomavirus self-sampling, chatbot, decision aid, health education, underserved populations, user-centered design, artificial intelligence, AI

## Abstract

**Background:**

Cervical cancer (CC) screening participation remains suboptimal among vulnerable populations in France. This study aimed to develop and evaluate AppDate-You, a chatbot-based decision aid, to support women from socioeconomically disadvantaged areas in the French Occitanie region to make informed decisions about CC screening, particularly human papillomavirus self-sampling (HPVss).

**Objective:**

This study aimed to explore the needs, preferences, and barriers related to CC screening and to design and validate a user-centered, empathetic, and effective chatbot-based decision aid to empower women experiencing socioeconomic challenges in France to make informed choices about HPVss.

**Methods:**

The chatbot was developed following a validated framework for developing decision aids. The process included qualitative research involving online and in-person interviews and focus groups with women and health care professionals, followed by alpha testing with both groups and beta testing with women only. Participants included women (both French and non-French speaking) aged between 30 and 65 years from socioeconomically disadvantaged areas of the Occitanie region and health care professionals (general practitioners, gynecologists, and midwives) working with these populations. AppDate-You was made accessible through WhatsApp and Facebook Messenger, offering text-based and voice-based interactions and multimedia content.

**Results:**

The exploratory phase identified key barriers to screening and digital tool preferences. Prototype testing revealed great satisfaction with the chatbot’s performance, educational value, and content quality. Contrary to the expectations of health care professionals, women from diverse backgrounds, including women who were older and socioeconomically disadvantaged, were willing and able to use the tool. Users—even those with limited digital literacy—found AppDate-You innovative, user-friendly, and informative. In the beta testing phase, 80% (12/15) of the participants expressed interest in HPVss. Some limitations were identified, such as the chatbot’s occasional repetitive responses and the need for clearer medical terminology.

**Conclusions:**

This study demonstrates the potential for artificial intelligence chatbots to improve access to health education and increase cervical screening intention among underserved populations. The user-centered approach resulted in a tool that effectively meets the needs of the target population.

**International Registered Report Identifier (IRRID):**

RR2-10.2196/39288

## Introduction

### Background

Cervical cancer (CC) remains a significant global health challenge, particularly among vulnerable and underserved populations with limited access to health care resources. While largely preventable through human papillomavirus (HPV) vaccination and regular screening, the effectiveness of interventions is heavily dependent on high participation and coverage rates. However, women experiencing socioeconomic challenges often face numerous barriers to accessing and participating in CC screening and prevention programs [1].

In France, an organized CC screening program was implemented in 2020, using HPV testing for women aged ≥30 years [2]. The program is available via various health care providers, including gynecologists, general practitioners (GPs), midwives, primary care facilities, and hospitals [2]. However, participation in CC screening programs remains suboptimal (59.5%), particularly among certain demographic groups [3]. Women aged >50 years, from socioeconomically disadvantaged backgrounds or underserved areas, or women with chronic health conditions, as well as women covered by supplementary health insurance, have notably lower screening rates [4].

Multiple barriers are responsible for suboptimal screening uptake. These include limited awareness of CC and its risk factors, insufficient understanding of screening benefits, inadequate access to health care services, cultural and linguistic barriers, fear or embarrassment associated with the screening process, and competing life priorities [5]. Addressing these multifaceted challenges is crucial to increasing participation rates and reducing disparities in CC prevention efforts.

To improve participation rates, the French government has issued national guidelines recommending a follow-up reminder at 12 months after invitation for women who do not respond to the initial screening invitation [6]. The reminder includes an HPV self-sampling (HPVss) kit, designed to overcome some of the barriers associated with traditional screening procedures.

A systematic review about values and preferences [7] reported evidence for the acceptability of HPVss regardless of age, income, or the country of residence. Two French randomized controlled trials (RCTs) [8,9] reported that mailing HPVss kits significantly increased participation rates compared to reminder letters alone. One study [8] reported an increase in uptake from 2% to 18.3%, while another study [9] showed an improvement from 11.7% to 22.5% [9]. Despite these promising results, it is important to note that <20% of nonrespondent women submitted the kit when mailed to their homes, indicating persistent barriers to participation [8,9].

There is growing recognition of the potential of decision aids to empower individuals to make informed choices about health care by providing them with evidence-based information [10]. Decision aids are designed to help people understand their different options and make informed decisions aligned with their values and preferences. Traditional educational methods often face limitations, such as inaccessibility, language and literacy challenges, and the inability to tailor information to individual needs [11].

In parallel with the evolution of decision aids, there has been increasing interest in leveraging technology to address health care disparities and improve access to medical information and services. Chatbots have emerged as a potential solution to engage patients and deliver educational content in a conversational format [12]. With their ability to provide 24-7 access to information, interactive and personalized guidance, as well as support, chatbots offer a promising solution to improve health literacy and patient engagement. Numerous artificial intelligence (AI)–based chatbots have been developed to provide cancer screening education, and several studies have demonstrated that users readily embrace these tools, which can serve as an additional source of information for individuals who may struggle to obtain or understand health-related guidance [13]. For example, chatbots have been developed to answer HPV vaccine–related questions [14], to improve colposcopy adherence after abnormal Pap test results in women living in underserved urban areas [15], or to promote HPV vaccination among girls in South Korea [16]. However, our chatbot is the first that aims to increase accessibility to information on cervical screening, with the option of self-sampling, targeted at women from a socioeconomically disadvantaged French area.

### Objectives

Considering the disparities in cervical screening uptake in France (as in many high-income countries) and the increased use of AI tools in health care, it is essential to explore how AI and the novel implementation of HPVss could improve equity in access to cervical screening. This paper focuses on the design and validation of a chatbot-based decision aid, specifically tailored to support women experiencing socioeconomic challenges in the Occitanie region of France to help them make informed decisions about CC screening. By leveraging qualitative research methods, we first aimed to explore the needs, preferences, and barriers faced by this population in relation to CC screening and then to create a user-centered, empathetic, and effective digital tool that can empower these women to make informed choices about HPVss.

## Methods

### Overview

**Figure 1:**
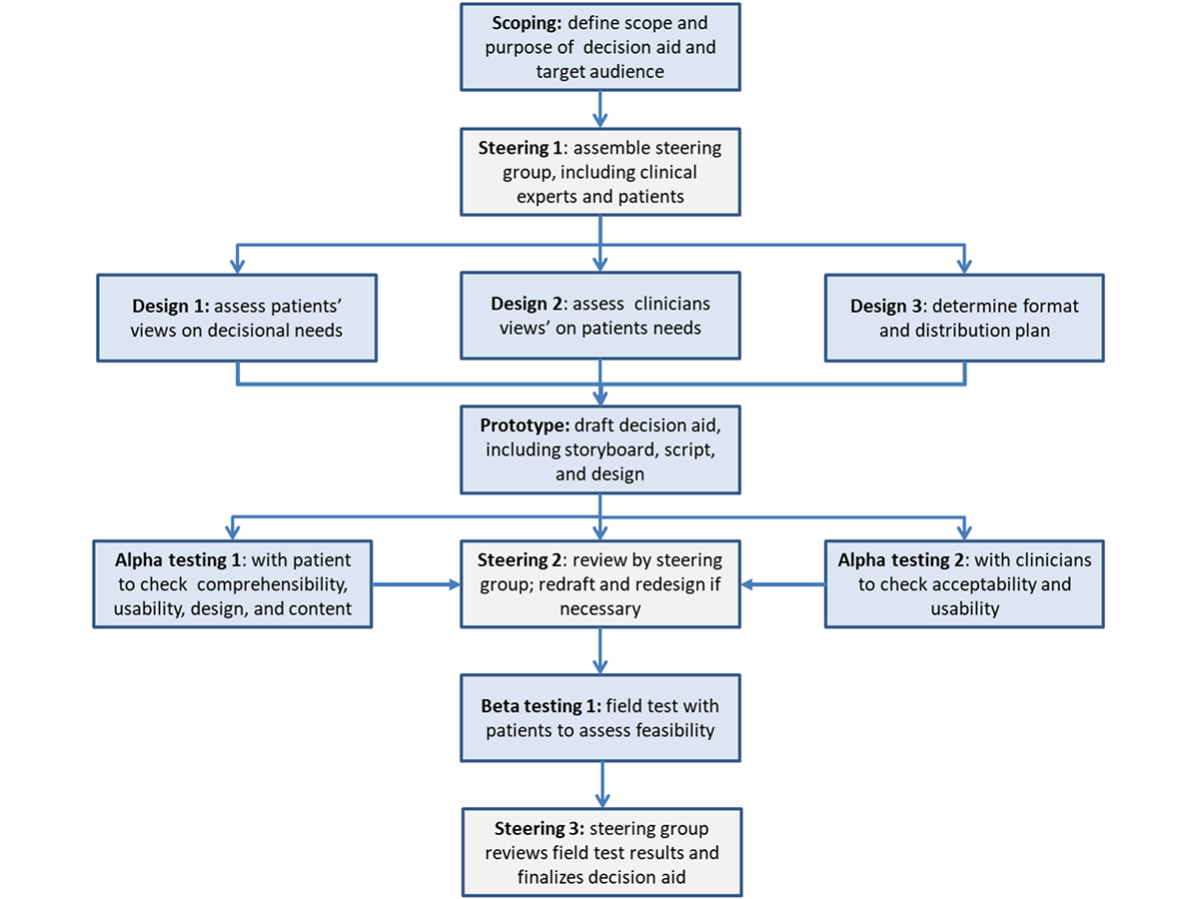
Model Development Process for Decision Aids (adapted from Coulter et al. 2013).

We followed the structured framework proposed by Coulter et al [17] for developing decision aids, based on the International Patient Decision Aid Standards, which consists of five key steps (Figure 1 [17]): (1) scoping the decision aid, (2) forming a multidisciplinary steering group, (3) designing the decision aid, (4) alpha testing (prototype testing), and (5) beta testing (field testing). We made slight adjustments to this framework to better suit our specific needs and context while maintaining its core principles. The design phase was meticulously structured to ensure that the decision aid was precisely tailored to meet the unique requirements and preferences of our target population. Notable modifications to the original framework include, first, the decision to exclude a literature review during the initial design phase, focusing instead on directly capturing the needs, preferences, and experiences of the women themselves. This decision was driven by time and resource constraints, as well as our commitment to a user-centered approach. Second, the decision to conduct beta testing exclusively with end users (eligible women), as the chatbot-based decision aid was designed for direct use without support from health care professionals. These adaptations improved the user-centered approach throughout the development process, ensuring that the final product would be highly relevant and accessible to our target population.

We organized the chatbot development process into 3 main operational phases in alignment with the steps proposed by Coulter et al [17], as described in Textbox 1.

Each phase was designed to iteratively refine the chatbot, ensuring it met the needs and preferences of our target population while adhering to the principles of effective decision aid development.

**Phases of the chatbot development process.**

- Phase 1: exploratory phase (September 2021 to November 2021)—this phase corresponded to the “scoping” and initial “design” stages of the framework proposed by Coulter et al [17]. We defined the tool’s purpose, established our multidisciplinary steering committee (comprising experts in screening, behavioral economics, and digital communication), and conducted qualitative research with eligible women and health care professionals to identify their needs, barriers, and preferences. The findings from this phase informed the initial design of the chatbot.
- Phase 2: prototype testing (June 2023 to July 2023)—this phase corresponded to the “alpha testing” step in the framework. We tested the chatbot prototype with both eligible women and health care professionals, gathering feedback on usability, content design, content comprehension, and acceptability.
- Phase 3: field testing (February 2024 to April 2024)—this phase corresponded to our adapted version of the “beta testing” step and was focused solely on end users. We tested the improved version of the chatbot with eligible women in real-world conditions to assess feasibility.

### Technical Development of the Chatbot

The AppDate-You chatbot was developed using the YeldaAI platform with a JavaScript-based architecture, integrating open-source large language models, such as Mistral and Llama, within a proprietary framework. This technical foundation enables natural language understanding, adaptive conversations, and secure hosting on certified Health Data Hosting servers in France, ensuring compliance with the European Union data protection standards.

The chatbot was built to be easy to access and use. Rather than requiring users to download a dedicated app, which participants in phase 1 identified as a potential barrier, AppDate-You was integrated with widely used messaging platforms—WhatsApp and Facebook Messenger. This approach leveraged existing platforms that most participants already had installed on their devices. Users accessed the chatbot by scanning a QR code that immediately initiated the conversation without requiring additional sign-up processes or authentication. Beyond text-based interaction, the chatbot can also process voice queries, adapting to diverse user preferences. Users can send voice messages through WhatsApp or Facebook Messenger, and the chatbot processes these messages and responds in both text and voice formats.

During its development, the project team conducted alpha and beta testing in collaboration with health care professionals and representative user groups. These tests informed refinements in conversational flow, language style, and tone, ultimately shaping a persona that represents a friendly, knowledgeable female health care figure. The deliberate choice of a female persona was based on input from our multidisciplinary steering group and target users, who indicated that women were likely to feel more comfortable discussing cervical screening with a female virtual assistant. Rather than adopting the role of a physician or an authoritative expert, AppDate-You presents itself as “Sarah,” a supportive peer from the medical field who can relate to the users’ experiences and concerns. This persona’s female voice was selected for its calm, reassuring tone that aligns with the chatbot’s overall empathetic design principle. The chatbot can also recognize emotional cues—such as worry or confusion—and responds with reassuring, plain-language statements before delivering factual details about screening procedures.

Privacy and data minimization were central considerations, reflecting the sensitivity of women’s health information. Users are advised not to share personally identifiable details, and any information provided inadvertently is automatically flagged or masked. All conversation logs remain on secure servers, accessible only to authorized personnel for maintenance or aggregated statistical analysis.

### Participant Recruitment

Purposeful sampling was used to select participants for the 3 study phases to ensure a representative sample, and those who participated in the first phase were excluded from subsequent phases of the study. This approach was implemented to obtain unbiased feedback throughout the iterative development process and to gather perspectives from a diverse range of women and health care professionals. We recruited women aged between 30 and 65 years residing in socioeconomically disadvantaged areas of the Occitanie region, France. The selection criteria included a range of sociodemographic characteristics: family status, employment status, country of origin, length of residence in France, French language proficiency, digital literacy, education level, and screening history. Recruitment was conducted by an external professional recruitment company and a local nongovernmental organization. Participants were recruited from existing panels, directories, or via the associative network. They were approached by email or telephone.

Health care professionals, including gynecologists, GPs, and midwives, were recruited from the Occitanie region. They were selected to represent diverse backgrounds, specialties, ages, years of experience, geographical locations, experience working with socioeconomically disadvantaged populations, experience in involvement in CC screening programs, and practice settings (eg, private practice, public hospitals, and public community centers).

The Occitanie region is the fourth most populous region in France, with 6.1 million inhabitants [18]. This region faces significant socioeconomic challenges, with an unemployment rate of 8.6% (compared to 6.9% nationally) and a poverty rate of 16.8% (vs 14.4% nationally), with marked disparities between departments [18]. To recruit women from socioeconomically disadvantaged areas, we used the Îlots Regroupés pour l’Information Statistique or Aggregated Units for Statistical Information classification of the French National Institute of Statistics and Economic Studies, which divides the territory into small geographic units of approximately 2000 residents with similar socioeconomic characteristics. These disadvantaged areas are characterized by high unemployment rates, a high proportion of manual workers, low average household incomes, and low education levels (low rate of high school graduates) [19].

### Data Collection

Data were collected by 5 interviewers (over the 3 phases) from an external company (BVA Xsight). All interviewers were women, had a master’s degree or a PhD in social sciences, and had previous experience in conducting qualitative research (from 1 to 13 years). The interviewers were employed by the external company as consultants, project managers, or managers of the qualitative studies unit.

### Phase 1

The objectives of the first phase were (1) to identify barriers faced by eligible women in participating in CC screening and their perceptions of HPVss, (2) to explore their smartphone use and perceptions of educational e-tools, (3) to identify difficulties faced by health care professionals in recommending CC screening to their patients, and (4) to explore the health care professionals’ perceptions of difficulties faced by their patients from socioeconomically disadvantaged backgrounds. An interview guide was developed based on these 4 objectives. The information gathered from this phase informed the development of the first AppDate-You chatbot prototype.

In total, 5 interviews (average duration: 1 h 30 min) and 2 focus groups (average duration: 3 h) were conducted in person with 15 non–French-speaking women in November 2021. The eligibility criteria included women from the Algerian or Georgian communities living in the city of Toulouse. Participants were recruited through associative networks. To anticipate language barriers, in each interview and focus group, a peer facilitator (a person from the community ensuring translation and understanding) was paired with the interviewer to support the discussion.

In total, 5 interviews (average duration: 1 h) and 2 focus groups (average duration: 2 h 30 min) were conducted online with 15 French-speaking women in November 2021. Eligibility criteria included women belonging to a working-class socioeconomic background, having a level of education no higher than high school graduation, and living in a socioeconomically disadvantaged area.

A total of 20 interviews (average duration: 1 h) were conducted over the phone or online with health care professionals between November and December 2021. Eligibility criteria included health care professionals working in the Occitanie area and being in contact with patients from socioeconomically disadvantaged areas or from ethnic minority groups.

### Phase 2

The aim of the second phase was to gather the opinions of women and health care professionals on the prototype, particularly on its acceptability, design and content, ease of use, and their understanding of the educational messages. An interview guide explaining this aim was provided to the participants.

Acceptability was defined as participant satisfaction and positive attitudes toward the chatbot experience, drawing on key constructs from the AI device use acceptance model [20,21]. We assessed this through structured interview questions focusing on ease of use (effort expectancy), user-interface satisfaction, content relevance and perceived usefulness and comfort (performance expectancy), and emotional responses to the interaction. These factors serve as strong indicators of whether women and health care professionals would use the tool.

Eligibility criteria were the following: belonging to a working-class socioeconomic background, having a level of education no more than 2 years beyond high school graduation, and living in a socioeconomically disadvantaged area. Eligible women could be either very comfortable using e-tools or not comfortable at all.

The interviews and focus groups were organized in 3 steps. First, preliminary phone interviews (approximately 20 min each) were conducted to assess women’s perspectives on health, CC screening, and smartphone use. Second, participants then engaged with the chatbot over a 5-day period, exploring specific topics related to CC. During this time, they were assigned 5 daily missions through an app designed to ensure comprehensive interaction with the chatbot’s content. Participants could also provide feedback and record their observations throughout the interaction period using this app. In addition, participants were encouraged to ask the chatbot further questions. Finally, in-depth interviews and focus groups were conducted to collect detailed feedback about their overall experience.

In total, 8 interviews (average duration: 1 h) and 4 focus groups (average duration: 2 h 30 min) were conducted online with 20 women (2 focus groups involved women who were comfortable with digital tools, and 2 focus groups involved women who were not comfortable with digital tools).

In total, 13 interviews (average duration: 1 h) were conducted with health care professionals (GPs, gynecologists, and midwives). Each participant was asked to assess the chatbot freely, with instructions sent by email.

### Phase 3

The aim of the third phase was to gather feedback from eligible women on the use of the chatbot (an improved prototype following the results of phase 2) and particularly to assess its feasibility in real-world conditions. The interview guide was developed to answer these key questions.

Feasibility was defined as a user’s ability to access and use the chatbot independently in real-world conditions. We evaluated this through (1) successful completion of initial access steps without assistance; (2) user-reported technical difficulties during the 5-day testing period; and (3) engagement metrics, including average session duration.

Participants were selected based on the following criteria: living in disadvantaged areas of the Occitanie region (specific areas selected based on socioeconomic level), not having participated in CC screening in the last 4 years, and being aged between 30 years and 65 years (the age range for CC screening eligibility in France).

A total of 15 eligible women were sent an invitation letter to participate in CC screening, including a guide explaining how to access the chatbot (via WhatsApp or Facebook Messenger). Women were encouraged to use the chatbot freely over a 5-day period. A facilitator was available to help access the tool if required. Following the testing period, participants were invited to an interview to share their experiences. In total, 15 interviews were conducted.

### All Phases

All interviews and focus groups were audio- or video-recorded with consent. Notes were also taken by the interviewers. Participants received financial compensation for their participation (between €30 and €50 [€1=US $1.183; approximate average for 2021] depending on the time spent). Interviewers had no previous relationships with the participants. Participants were informed both in writing and verbally about the aim of the research, as well as the data collection and analysis process, and were asked to sign an informed consent form beforehand. Participants were informed that the interviews and focus groups were conducted as part of a research project aiming to understand their needs and expectations in terms of quality of life and health. The interviews and focus groups were organized online or in person. When in person, they were organized in private dedicated spaces or rooms belonging to the associative network. Besides the participants and the researchers, only the peer facilitators were present when required. Interviews and focus groups were conducted until enough data were obtained.

### Ethical Considerations

This study was approved by the International Agency for Research on Cancer ethics committee (IEC 21-16). All methods were performed in accordance with the relevant guidelines and regulations outlined in the Declaration of Helsinki. All participants provided written informed consent after being informed about the study’s aims and procedures. For non–French-speaking participants, a peer facilitator assisted with translation to ensure comprehension of the consent process.

All data were anonymized during analysis. Recruitment information, contact details, audio recordings, and interview transcripts were stored on secure servers with access restricted to the research team and automatically destroyed within 3 months after completion of each study phase. For chatbot testing phases, conversation logs were secured on certified Health Data Hosting servers in France, complying with the European Union data protection standards, and destroyed after completion of analysis for each phase. Users were advised against sharing personal identifiable information during chatbot interactions, and any personal details inadvertently shared were masked in the analysis.

Participants received financial compensation ranging from €30 to €50 (€1= US $1.183), depending on the duration of their participation, to compensate for their time commitment and potential travel expenses.

### Data Analysis

The interviews and focus groups were transcribed manually. For non-French recordings, the transcription was completed with the assistance of peer facilitators who participated in the interviews. These peer facilitators provided translation during the interviews and assisted with the translation of recordings during transcription to ensure accurate representation of participants’ responses in French while preserving cultural context and meaning. An initial content analysis was performed manually following the method described by Braun and Clarke [22]. This initial analysis was performed by the external supplier who conducted the interviews, involving 3 data analysts who had expertise in qualitative research. The analyses were then reviewed and validated by a senior behavioral economics expert from the external company. An analysis grid was preliminarily developed to categorize participant feedback into themes and subthemes. The focus was placed on the barriers, facilitators, attitudes, and motivations toward CC screening, particularly HPVss, as well as gathering the insights, views, and recommendations from women and health care professionals to enhance the chatbot’s design and content. A subsequent deductive analysis was then performed by the first author (ALB) to reorganize identified themes to match our objectives. This second analysis involved selecting the data to inform the key aspects of the chatbot’s development, that is, views on HPVss, experiences with the tool, and perspectives on its use.

Participants in the focus groups were organized into numbered groups with different criteria across phases. In phase 1, a total of 4 focus groups were organized by language background: groups 1-2 French-speaking women and groups 1-2 non–French-speaking women. In phase 2, four focus groups were organized by digital comfort level: groups 1 and 3 included women who were not comfortable with digital tools, while groups 2 and 4 included women who were comfortable with digital tools. For individual interview participants, pseudonyms (eg, Esther and Eric) were assigned to protect anonymity while enhancing the readability of direct quotations, in accordance with standard qualitative research practice.

### Validity Procedure

The methods used follow recommendations about the validity of results [23]. Credibility was satisfied through the implementation of several systematic procedures throughout our research process. All interviews and focus groups were conducted using standardized guides to maintain consistency across data collection. For non–French-speaking participants, peer facilitators helped translate discussions to ensure accurate understanding across languages. Data triangulation was achieved by comparing perspectives across different participant groups (French-speaking women, non–French-speaking women, and health care professionals).

Authenticity was satisfied by the achievement of interviews and focus groups until a sufficient diversity of perspectives was captured and data saturation was reached. Moreover, our 3-phase iterative development approach served as an additional validation mechanism, as participant feedback directly informed subsequent improvements to the chatbot.

Concerning transferability, comprehensive and detailed explanations about the procedure of the interviews and focus groups have been provided, and the interview and focus group grids (in French) are available upon request.

Finally, concerning confirmability, we used 3 analysts to code the thematic analysis. Investigator triangulation was used through a team-based analytical approach, where multiple researchers from the external company independently coded transcripts before meeting to reach consensus on themes. Regular meetings between our research team and the external company ensured alignment with research objectives throughout the data collection and analysis process.

## Results

Participant sociodemographic characteristics are provided in Tables S1-S6 in Multimedia Appendix 1.

### Phase 1: Exploratory Phase

#### Views on HPV Self-Sampling

##### Health Care Professional Perspective

###### Self-Sampling Is Easy

Self-sampling was considered simple, easy, and accessible to all women, and without risk of harm or injury to oneself. Self-sampling was perceived as ideal for women with an aversion to gynecological examinations (due to embarrassment). Most health care professionals considered self-sampling to be in line with present-day practice, as people are used to self-sampling with COVID-19 tests:

No, women know how to insert tampons, women clean themselves far too much, as I told you, and far too often on the inside, it’s child’s play.

Esther, GP

###### Self-Sampling Might Not Be Appropriate for All Women

However, health care professionals reported that self-sampling would not be suitable for all women, specifically women who do not know their own anatomy and women who do not participate in self-testing in general. Moreover, they assumed that older women (regardless of their backgrounds) would not adhere to this procedure:

Let’s say that the self-test is mainly intended for patients who are already comfortable with their bodies and who are, for example, accustomed to using tampons or menstrual cups or inserting things internally.

Sophie, midwife

A lack of interest or willingness among women from socioeconomically disadvantaged areas toward screening campaigns in general was also mentioned by health care professionals:

It’s generally these women who throw away the mail and who don’t really care about it.

Morgane, midwife

###### Self-Sampling Could Deter Women From Consultations

All health care professionals reported that the self-test was a good screening tool to improve screening participation, but it should not replace a clinical examination or the Pap smear test. Instead, self-testing should complement regular follow-up. Some health care professionals perceived the self-test as a competitor and feared that patients would skip necessary clinical examinations:

The concern is that they won’t have a clinical examination. ‘It’s ok, I did it at home, it’s fine,’ it shouldn’t become an excuse to get out of doing it.

Hélène, gynecologist

##### Women’s Perspectives

###### Self-Sampling Can Improve Access to Screening

The women did not anticipate any particular difficulties and felt comfortable with the idea of self-sampling. The self-sampling test appeared simple, with only a few steps, and the instructions were considered sufficient. Handling the cotton swab was reported as familiar, as many women had already done a polymerase chain reaction test for COVID-19. The main advantages of HPVss highlighted by women were being reassured between gynecological examinations, saving time, having a solution when it is difficult to consult a specialist, being able to do the test comfortably at home (less stressful), and having an alternative for when they did not want to consult a male health care professional for gynecological procedures:

It’s good for women who work all day because we don’t have much time.

Zourika, aged 35 y, non–French-speaking woman

Living in Hérault as well, there’s an issue, anything gynaecology-related is quite complicated, very long to get appointments, so yes, I support this kind of proposal, and if it really replaces the Pap smear, there’s no need to go to the gynaecologist anymore, at my age, she just gives me a Pap smear, sends me the results, and that’s it. It can free up places for people who need to be monitored, it’s positive.

French-speaking woman, group 2

###### Self-Sampling Can Be Perceived as Complex

For all women, several obstacles could hinder adherence to self-sampling. First, the complexity of the test was often considered too technical. Introducing the cotton swab into the vagina and how to correctly collect a sample before removing it was considered very technical and required expert hands:

I don’t know if I would do the test, and I’m even rather against it because I prefer to have it done by a specialist because I don’t know if I would do it correctly, if I would know what to do to get an accurate result.

Madina, aged 32 y, non–French-speaking woman

Second, the fear of hurting themselves was also mentioned by the participants. The test was not perceived as a pleasant moment, and having it carried out by another person seemed to make things easier. However, for some participants, having the responsibility of conducting an unpleasant test on themselves was too demanding:

I would also be afraid of hurting myself, of inserting it in the wrong place or damaging something.

Sophie, aged 39 y, French-speaking woman

###### Self-Sampling Is Perceived as Unreliable

Concerns were raised about the reliability of the self-sampling test, as self-sampling is not as deep as samples taken by a health care professional using a speculum. In addition, the risk of a false negative result if the sample was not taken correctly was also expressed by the participants:

I would be more inclined to do the first two steps, for the third one I would be afraid of not turning it correctly, I wouldn’t trust the result. I’m not convinced. It’s the act of inserting it into the vagina and turning it. If they tell me it’s positive, I’ll consult immediately, if they tell me it’s negative, I wouldn’t be convinced.

Magali, aged 48 y, French-speaking woman

Participants who had regular gynecological follow-up indicated their clear preference for screening to be performed by a health care professional:

I prefer to have my doctor do the smear. I don’t feel confident doing it myself. I’d rather have a professional who’s used to doing it, who knows how to do it, who knows how to do it properly. I would be more comfortable with the results.

Sophie, aged 39 y, French-speaking woman

###### Women Could Miss Out on the Benefits of a Consultation

Without medical supervision, women with positive results might not follow up on additional examinations. The risk of replacing gynecological follow-up with self-testing was highlighted, as some women interested in self-sampling mentioned the possibility of no longer going to their physician:

I don’t think so, because I have regular check-ups and I tend to bury my head in the sand, what I don’t know can’t hurt me. I wouldn’t do it in addition to my regular check-ups. For those who have difficulty finding a practitioner, it might be a good alternative. I don’t think it would be necessary in my case, but it could be useful for other people.

French-speaking woman, group 1

#### Perspectives on the Chatbot Platform

##### Overview

For both health care professionals and women, the scope of the app was not broad enough. One would not need to consult it often for its current purpose of just CC screening. Consequently, downloading the app did not seem worthwhile to participants:

It’s a good idea, but would it be an application just for that? An application seems odd, I don’t think people will download it just for that. It could be an application but for screenings for different types of cancers, then I’d say yes, the scope is broader.

Marie, aged 59 y, French-speaking woman

##### The Health Care Professional Perspective

###### Limited Interest in e-Tools in General

Health care professionals expressed limited interest in e-tools in general, but they reported a growing interest among their patients:

Often, they talk about it, ‘Oh, I have an app, doctor, I’m going to check it,’ and it irritates me.

Hélène, gynecologist

###### Health Care Professionals’ Preconceptions About Digital Tool Use

Health care professionals expressed some preconceptions about older women and women from disadvantaged areas who might not be interested in e-tools or would not be able to use them:

But if you like, the problem with sites is that the people who need it most, if you want to understand things properly, are often people who don’t often have a computer or who are a bit limited, you know what I mean?

Anne Marie, GP

However, the development of the app and the use of HPVss was supported by all health care professionals, on the following conditions:

- It is specified that a self-sampling test cannot replace a test performed by a health care professional or a clinical examination.
- The app includes a personalized reminder with support mechanisms in place in the event of a positive result.
- The app is extended to other screening programs and includes reminders, information on the screening program, and directories to help women find health care professionals.

##### User Perspective

###### Use of the Internet and Smartphones in Daily Life

The women interviewed had at least one smartphone for personal use and most also had at least one laptop. If they had technical problems, they reported no hesitation in asking their children to help them navigate or configure their devices. Non–French-speaking women, who were not familiar with the term “app,” confused it with the social media website pages:

I use my smartphone for everything, I make my medical appointments, do my shopping list and my online shopping, searches for myself or for the little one’s homework, I go on social media.

French-speaking woman, group 1

Strong interest in the app and HPVss was expressed by non–French-speaking and older women, who had not consulted a gynecologist for a long time. Less interest was expressed by younger and French-speaking women who preferred to consult their physician directly:

I think it’s great, a super idea. In Hérault, it is very difficult to get an appointment, it’s ridiculous in Montpellier, I have to wait 6 months. We don’t have regular follow-ups like we used to have 15, 20 or 30 years ago, now we just go for a Pap smear, so if we can do it at home with the time we need to do it, it’s great to be able to do it ourselves.

non–French-speaking woman, group 2

All women showed interest in the project to develop the e-tool and use HPVss, provided the following:

- The e-tool is recommended by a trusted source (eg, physician, friend, and family member) or a reliable authority (eg, French Health Insurance).
- The self-sampling test is free of charge, sent to the home with a prestamped return envelope, and results are easily and securely accessible.
- The e-tool provides audio-video tutorials on how to do the self-sampling test, as well as information about cancer, screening, and prevention in the user’s preferred language.
- The e-tool provides information beyond CC screening, including broader health prevention topics.
- The e-tool is integrated into commonly used social media platforms for easy access and sharing.

###### Limited Interest Among Some Women

Some participants reported that they would prefer to consult their health care professional directly, as they were more comfortable doing so and testing performed by a health care professional was more reliable. These women were French-speaking and benefited from regular follow-up:

The only thing I’d be interested in is having some kind of follow-up to reminds us, ‘Don’t forget, it’s been 11 months since your last screening, it would be good to make another appointment.’ Otherwise, I don’t really see the point. If I want information about the disease, I’d rather talk about it with my gynecologist or my GP.

French-speaking woman, group 1

### Phase 2: Prototype Testing

#### Overview

The second phase of the study aimed to gather feedback from health care professionals and women on the prototype. Participants provided feedback on the acceptability, design and content, ease of use, and understanding of the educational messages. The AppDate-You chatbot was made available through WhatsApp, offering text-based and voice-based interactions.

#### User Experience and Interface Design

All participating women provided positive feedback on the chatbot’s performance, highlighting its speed, precision, and reliability in delivering responses. Notably, even participants with limited interest in smartphone apps found the tool accessible and user-friendly. The inclusion of a vocal option was particularly appreciated, enhancing the chatbot’s usability. The integration with WhatsApp emerged as a key feature, praised by both women and health care professionals. Health care providers specifically noted the advantage of using WhatsApp as a platform, citing its free availability and widespread adoption among the target population. This choice of platform seemed to remove barriers to accessing the tool and increase the chatbot’s potential reach:

Very easy, even though I’m usually not very good smartphones, computers, etc., but this was easy.

Hayat, aged 42 y

Despite the generally positive reception, usability testing revealed some challenges. A key issue reported by some women was the need to frequently rephrase questions to obtain relevant answers from the chatbot, leading to frustration and an impression of time-wasting. Users also expressed dissatisfaction with repetitive responses, particularly when the chatbot failed to understand queries or lacked appropriate information:

A question or two where I had to insist, age-wise, it always gave me the same answer, and then I got no video, no link to a site.

Christine, aged 45 y

#### Educational Value and Content

All participating women reported increased knowledge from using the chatbot, particularly about self-sampling, screening, HPV, and HPV vaccination. Participants expressed interest in using similar tools in the future, highlighting the importance of health care professional validation. Health care professionals agreed with its educational impact, praising the relevance and clarity of the information provided. They described the chatbot’s answers as scientifically accurate and easily understandable, highlighting its potential as an effective tool for health education:

It’s interesting, even fun to have the answers, to be able to chat like that. And so I think it might interest people to have a quick and reliable answer.

Eric, GP

I would love to use it, especially if you create an app like this, make sure you say that it’s verified by healthcare professionals, it is really important.

Chehrazade, aged 35 y

Health care professionals appreciated that the chatbot appropriately guided users to seek in-person medical consultations when necessary:

Frankly, he can give them information quickly [...] he tells them to go and consult, when to consult a professional, no, frankly I think it can be interesting.

Olivia, midwife

Users valued the freedom to ask various questions without feeling embarrassed or judged:

Sometimes it can be embarrassing to ask a professional this kind of question.

Maryline, aged 36 y

I would say discretion. Sometimes some people are afraid of what other people will think.

woman, group 1

Users also praised the chatbot’s flexibility in handling concise queries regardless of spelling or grammatical errors. The inclusion of links to informative videos was noted as a particularly useful feature, enhancing the educational experience. Importantly, women suggested simplifying complex and technical information through the use of images, illustrations, and infographics, highlighting a preference for visual learning aids to enhance understanding of medical concepts:

A diagram yes, that’s right, that would have been good.... A diagram would be great.

Chehrazade, aged 35 y

Both: testimonials and images like we can see in the photo, with drawings, showing the progression, small diagrams.

Hayat, aged 42 y

#### Feedback on HPVss-Related Content

While all women were not aware of HPVss before participating in the study, they all showed a good understanding of the benefits of using HPV self-sampling. This positive feedback was shared by the health care professionals, who mentioned that the HPVss was presented well in the chatbot. However, both women and health care professionals highlighted a lack of clarity for the following aspects. First, the price of a self-sampling kit and reimbursement rates. Second, how to obtain a self-sampling kit (eg, availability in pharmacies, availability online, and necessity for a prescription):

I had trouble understanding where you can get the self-test. It said on the Internet. This is not reassuring.

Woman, group 3

Third, women were concerned with the lack of clarity about the kit’s delivery conditions and (eg, protection of the tube against temperatures, packaging, and deadline to send it) and communication of the test results (eg, confirmation of receipt and waiting times). Fourth, health care professionals were concerned about a lack of information about screening follow-up modalities depending on test results. Finally, women were concerned about the lack of success stories or shared experiences from other women having used a self-sampling kit, which would serve to reassure them.

In addition, women found that the e-tool put too much emphasis on HPVss in the presentation of screening procedures for CC. They advised that it should be moderated. Health care professionals also insisted on the fact that self-sampling is only one way to get screened and that even if women opt for self-sampling, they should still consult their gynecologists regularly:

It felt like it wanted to sell us the self-test.

Woman, group 3

That’s all very good, but I think that if all women received their kit at home, they would no longer go to see the gynecologist.

Léonie, midwife

Finally, the video presenting the HPVss kit was much appreciated by both women and health care professionals. This video was developed by another French research team working on promoting HPVss. Participants found the 4-minute, 15-second video to be easy to understand, accessible to all, and sufficiently detailed. Health care professionals reported that this format caught their attention and encouraged them to recommend self-sampling to their patients. Some women even mentioned that they learned more about their anatomy while watching the video:

It is interesting because it can help people have a better understanding of their internal anatomy.

Woman, group 4

However, both women and health care professionals suggested improvements to the video (shortening its length and making it more engaging) and adding step-by-step illustrations of the screening process. Moreover, due to inconsistent display of the video link, some participants never saw the video and consequently lacked understanding of how to use the self-sampling kit:

It was too long, you know. I lost interest, I didn’t follow all the way to the end.

Olivia, midwife

#### Accessibility and Target Audience

Some health care professionals expressed concerns about the chatbot’s reach and target audience. They were worried that the tool might not be accessible to women experiencing socioeconomic disadvantage, especially those with low literacy or without smartphones. They also felt that the tool would only be of interest to young women (aged <30 y) who are comfortable using smartphones, although this concern was not reported by the participating women themselves:

For my underserved patients, they don’t necessarily have smartphones, so it won’t reach everyone. And I have patients who can’t read, gypsies for example.

Marjolaine, midwife

### Phase 3: Field Testing

#### Overview

The third phase evaluated the improved AppDate-You chatbot prototype for usefulness, feasibility, and acceptability among eligible women. An enhanced version based on phase 2 feedback was made accessible via WhatsApp and Facebook Messenger. This version incorporated rich multimedia content, including illustrations and infographics, covering various aspects related to CC and screening procedures. We also created a new, more concise video explaining how to perform HPVss that was informed by participant feedback from phase 2 and addressed the concerns about length and monotony. This improved visual approach aimed to further simplify complex information and enhance user understanding.

#### User Experience and Use Patterns

Most participants provided positive feedback on the chatbot’s use. In total, 5 participants expressed difficulties accessing the chatbot, and 1 participant requested help from the facilitator to access it. Overall, 93% (14/15) of the participants were able to access the chatbot independently. Participants found the tool easy to use and compatible with their usual online communication platforms, particularly WhatsApp. Interactions with the chatbot were consistently described as straightforward. On average, participants engaged with the tool for about 10 minutes daily, asking between 1 and 3 questions per session. Notably, the topic that generated the most interest among users was the HPVss test:

The tool is easy and quick, you don't need to go on forums, look for something related to a very specific issue, and the answers are there, instantaneously, and useful. Like the links.

Nadhira, aged 58 y

Despite overall positive feedback, some limitations were noted. A recurring issue was user frustration with the chatbot’s tendency to provide repetitive answers to some queries:

Minor reproach: There are too many unnecessary images. It’s always the same ones over and over again.

Claudine, aged 45 y

#### Educational Value and Content Quality

Participants appreciated the chatbot’s content, describing it as diverse, informative, clear, and useful. They reported significant knowledge gained while using the tool, particularly about CC screening:

Really useful, I've learned things I didn't know, it's made me want to go for a smear test, although I wasn't aware of it, but now I know why I should do it, and how to go about it. And if it prevents me from getting cancer, it is important.

Sonia, aged 30 y

The chatbot effectively raised awareness about CC screening, but some limitations were identified. Users found that the chatbot’s answers sometimes did not sufficiently encourage or prompt users to delve deeper into the topics discussed. Moreover, the use of medical terms, particularly abbreviations, occasionally caused confusion. For example, one participant had to search online for the meaning of “HPV,” highlighting the need for clearer explanations of medical terminology.

#### HPVss Perceptions and Impact

The final test revealed a positive perception of the information on HPVss. Moreover, 80% (12/15) of the participants expressed interest in HPVss when it becomes available. Users perceived HPVss to be a safe, private, easy, and reliable method that provides fast results:

It’s very practical, it’s reassuring to do it at home, it saves time and it makes it easier for many people to get screened and encourages them to do it.

Ophélie, aged 38 y

The educational video demonstrating the HPVss steps helped to change women’s attitudes toward screening. Several women who were initially hesitant about self-sampling reported feeling more confident after watching the demonstration:

It’s reassuring because it shows that tests done at home are just as reliable as if they’d been done at the doctor’s. They show you how to do it, what position to adopt and, above all, you have to be very relaxed for the test to be carried out properly. And once you’ve taken the test, you have to send it the same day, by post, to get the results within 15 days.

Shalini, aged 32 y

The video is really important, without it the subject seems a bit unclear.

Magali, aged 50 y

Self-sampling was seen as a potential solution to various barriers, including difficulties in obtaining health care appointments, past negative experiences with professionals, and discomfort associated with examinations performed by professionals:

I think it’s good for people like me who refuse to go to the doctor.

Michelle, aged 61 y

Three women preferred not to use self-sampling due to a lack of confidence in performing the test correctly themselves, fear of self-injury, or a general dislike of self-tests. However, all participants recognized the value of self-sampling for those with limited access to health care or time constraints.

## Discussion

### Principal Findings

This paper outlines the design and validation phases of a chatbot designed to help women from socioeconomically disadvantaged areas in France to make an informed choice about cervical screening. Our multiphase approach ensured AppDate-You’s development as a user-centered, empathetic, and effective digital decision aid. The user-centered design evolved throughout our iterative process that continuously incorporated women’s feedback. For example, we added additional videos and infographics in response to suggestions to simplify complex medical information. Women in phase 3 particularly appreciated these user-centered adaptations and found the visual content especially helpful to understand screening procedures. We also removed technical barriers for users with varying digital literacy levels by integrating with widely used messaging platforms and offering voice interaction options. Empathy was achieved through conversational tone and a nonjudgmental approach. Women appreciated the chatbot’s ability to provide a comfortable environment to freely ask questions without feeling embarrassed or judged. The effectiveness of AppDate-You was demonstrated through dual validation: users reported substantial knowledge gained and increased likelihood of screening uptake, while health care professionals confirmed the content’s scientific accuracy and appropriate guidance in phase 2.

Findings from phases 2 and 3 also demonstrated high acceptability and feasibility of the AppDate-You chatbot among the target population. Acceptability was shown by feedback from both participants and health care professionals on the chatbot’s performance, educational value, and content quality across testing phases. Users appreciated its accessibility through familiar messaging platforms, user-friendly interface, and ability to provide scientifically accurate information. Regarding feasibility, most participants accessed and navigated the chatbot independently without technical assistance, engaging with the tool for approximately 10 minutes on a daily basis, with HPVss emerging as the topic that generated the most interest and questions among users. Beyond evaluating the chatbot itself, this research also provided valuable insights on the acceptability of HPVss in France. While the HPVss procedure was perceived as easy and approachable by both health care professionals and the participants, some challenges were identified by participants. Some women mentioned a lack of confidence in using the device correctly and questioned its reliability. Health care professionals expressed a fear of deterring some patients from consultations.

The AppDate-You chatbot was designed to assist women in making informed decisions about HPVss and has garnered positive feedback from both users and health care professionals. Notably, even individuals with limited digital literacy or minimal familiarity with AI found the chatbot to be innovative, user-friendly, and highly informative. Users particularly appreciated the intuitive interface, rapid response times, and the quality of information provided. The incorporation of multimedia elements, such as infographics and videos, proved effective in simplifying complex health concepts and enhancing overall understanding of HPV and CC screening.

### Comparison to Previous Work

Our findings showed that the chatbot not only enhanced knowledge about CC screening but also significantly boosted motivation to engage in screening practices. These results underscore the potential of digital interventions to advance and promote health education, especially among populations less engaged in preventive health practices. These promising outcomes are consistent with recent studies, notably the study conducted by Baumgärtner et al [24], which demonstrated that the PROState cancer Conversational Agent chatbot for prostate cancer screening achieved high user engagement and satisfaction while effectively conveying health information and improving understanding of screening processes.

In recent years, chatbots have been increasingly used across the spectrum of oncological care, from early prevention and screening to patient support during treatment for various types of cancer [12]. Evaluations consistently report high levels of user satisfaction and have shown that these tools are effective in key areas, including enhancing patient-centered communication, improving access to cancer-related information, and facilitating better overall engagement with oncological services [12].

Chatbots can be delivered through various channels, with WhatsApp emerging as a particularly valuable platform for health education. Pereira et al [25] demonstrated that WhatsApp-based interventions significantly enhance breast cancer knowledge and serve as a viable alternative to support breast cancer control strategies [25]. Similarly, in our study, WhatsApp was highly appreciated and widely used by the participants, proving to be more accessible than Facebook Messenger. This preference is likely due to WhatsApp’s widespread adoption and the greater familiarity it holds among our target women.

Our results are in accordance with international evidence, showing that HPVss is generally acceptable to women, but a lack of self-confidence with collecting a reliable sample can be a barrier to choosing this option [7]. A study conducted in France showed that health care professionals have positive attitudes toward HPVss**.** More precisely, health care professionals indicated that self-sampling could help to remove logistical, organizational, financial, and psychological obstacles [26]. Our study demonstrated similar results; however, our participants also expressed concern that self-sampling could deter women away from consultations and might not be suitable for all women.

Given the current lack of standardization in the development and evaluation processes for chatbot health care tools [27], we adopted the framework proposed by Coulter et al [17] to design our chatbot. This comprehensive approach consisted of 3 key phases: identifying CC screening information needs, conducting user testing, and implementing iterative improvements. By adhering to this systematic process, we significantly enhanced the chatbot’s performance and aligned our tool with women’s needs before its widespread implementation. The beta testing phase resulted in only minor revisions, indicating the robustness of our initial design and the value of behavioral science and our user-centered approach.

### Strengths and Limitations

A notable strength of our study is the thorough approach we adopted to collect opinions and feedback from both women and health professionals. We used a validated framework to develop a chatbot and a range of methods tailored to the specific objectives of each phase, including face-to-face and online interviews and focus groups. This diverse methodological approach ensured a comprehensive understanding of user experiences and preferences, enhancing the robustness and depth of our findings. However, while the sample size was adequate to gather feedback and capture perceptions, it may not be fully representative of the broader population of women in disadvantaged areas of France. We also acknowledge that digital health interventions may not reach all demographic groups equally. Certain racial, cultural, or gender groups might be less likely to engage with AI-based health tools due to technological barriers, language limitations, or cultural preferences for in-person health care. To mitigate these barriers, we implemented several design choices: (1) creating a female virtual assistant named “Sarah,” an internationally recognizable name acceptable across many cultures; (2) offering both text and voice interaction options to accommodate various literacy levels; and (3) ensuring the language used was simple and accessible. Despite these efforts, we recognize that some groups may still be underrepresented among users, highlighting the importance of complementary approaches when promoting CC screening.

### Future Directions and Implications

A key finding of our study is the divergence between health care professional expectations and women’s actual experiences with the chatbot. While professionals anticipated that older women and those from underprivileged backgrounds would struggle with or show disinterest in e-tools, the reality was quite different. These women demonstrated both willingness and ability to engage effectively with digital health solutions. This difference suggests that health care professionals may underestimate patients’ ability to engage with and benefit from digital health tools. Several factors could contribute to this misconception. First, health care professionals might be influenced by stereotypes about age and socioeconomic status in relation to technology use. Second, they may lack up-to-date information on the increasing digital literacy across diverse demographics. In addition, the user-friendly design of the chatbot, with its clear interface and engaging content, may have played a crucial role in overcoming potential barriers. These findings have important implications for the development and implementation of digital health solutions. They underscore the need for health care providers to reassess their assumptions about patient capabilities and to involve diverse user groups in the design and testing phases of digital tools.

While AI chatbots show promise in increasing health knowledge and enhancing motivation to adopt health-related behavioral changes, robust RCTs are still needed to draw definitive conclusions about their effectiveness in health care [28]. We plan to conduct a 2-arm cluster RCT to evaluate the impact of our chatbot-based decision aid on CC screening participation rates. The detailed study protocol has been previously published [29]. This trial will provide valuable empirical evidence on the real-world effectiveness of digital interventions in promoting preventive health care behaviors and help to establish their role in improving health outcomes.

In conclusion, our study highlights the potential for AI chatbots to improve health education and promote cervical screening among underserved populations. By leveraging user-friendly digital tools integrated into widely used platforms, we can address potential barriers and enhance health outcomes. Future research, particularly well-designed RCTs, will be essential to validate these findings and refine the implementation of digital health interventions. Our planned RCT will be a critical step toward providing evidence on the impact of chatbot-based interventions on CC screening rates and will contribute to the broader understanding of digital health tools in preventive care.

## Appendix

Multimedia Appendix 1 Participant sociodemographic characteristics.

## Abbreviations

AI: artificial intelligence
CC: cervical cancer
GP: general practitioner
HPV: human papillomavirus
HPVss: human papillomavirus self-sampling
RCT: randomized controlled trial

## Data Availability

The data that support the findings of this study are available upon request from the corresponding author. The data are not publicly available due to privacy and ethical restrictions.
