# Supplementary material for: Design and Validation of a Chatbot-Based Cervical Cancer Screening Decision Aid for Women Experiencing Socioeconomic Disadvantage: User-Centered Approach Study
Source: JMIR Cancer. 2025 Jul 24;11:e70251. doi: 10.2196/70251 (PMC12332448; doi:10.2196/70251)
Supplement: Multimedia Appendix 1 [file cancer_v11i1e70251_app1.docx]

**Table S1:** Non-French speaking women characteristics – Phase 1 participants

| **Demographics** | **Categories** | **Number of participants  (total = 15)** |
| --- | --- | --- |
| Age |  |  |
|  | 30-45 years old | 8 |
|  | 45-55 years old | 4 |
|  | 55-65 years old | 3 |
| Family status |  |  |
|  | Married with children | 14 |
|  | Separated with children | 1 |
| Country of birth |  |  |
|  | Armenia | 5 |
|  | Algeria | 5 |
|  | Georgia | 2 |
|  | Chechnya | 2 |
|  | Tunisia | 1 |
| Professional status |  |  |
|  | Housewife | 6 |
|  | Employed | 5 |
|  | Waiting for work approval documents | 2 |
|  | Looking for employment | 1 |
|  | Retired | 1 |
| Education level |  |  |
|  | Elementary school | 4 |
|  | Middle school | 4 |
|  | High school | 6 |
|  | No school | 1 |
| Date of arrival in France |  |  |
|  | Less than 10 years ago | 6 |
|  | More than 10 years ago | 7 |
|  | More than 20 years ago | 2 |
| Participation in cervical cancer screening |  |  |
|  | Never/ does not remember | 12 |
|  | At least once | 3 |

**Table S2:** French-speaking women characteristics – Phase 1 participants

| **Demographics** | **Categories** | **Number of participants**  **(total = 15)** |
| --- | --- | --- |
| Age |  |  |
|  | 30-45 years old | 6 |
|  | 45-55 years old | 7 |
|  | 55-65 years old | 2 |
| Family status |  |  |
|  | Single | 3 |
|  | Single with children | 3 |
|  | In a relationship with children | 5 |
|  | In a relationship with adult children | 4 |
| Area |  |  |
|  | Hérault | 4 |
|  | Haute-Garonne | 3 |
|  | Pyrénées-Orientales | 3 |
|  | Gard | 2 |
|  | Aude | 1 |
|  | Ariège | 1 |
|  | Gers | 1 |
|  | Tarn | 1 |
| Professional status |  |  |
|  | Employed | 9 |
|  | Looking for employment | 1 |
|  | Housewife | 2 |
| Education level |  |  |
|  | High school | 9 |
|  | High school graduation + 1 or 2 years | 4 |
|  | Professional degree | 1 |
|  | No school | 1 |
| Participation in cervical cancer screening |  |  |
|  | Never/ does not remember | 11 |
|  | At least once | 4 |

**Table S3:** Healthcare professionals characteristics – Phase 1 participants

| **Demographics** | **Categories** | **Number of participants**  **(total = 20)** |
| --- | --- | --- |
| Age |  |  |
|  | Less than 45 years old | 5 |
|  | Between 45 and 60 years old | 7 |
|  | More than 60 years old | 8 |
| Speciality |  |  |
|  | General Practitioner (GP) | 10 (5 women/ 5 men) |
|  | Gynaecologist | 4 women |
|  | Midwife | 6 women |
| Area of practice |  |  |
|  | Hérault | 6 |
|  | Haute-Garonne | 4 |
|  | Pyrénées-Orientales | 3 |
|  | Aude | 2 |
|  | Gard | 2 |
|  | Ariège | 1 |
|  | Gers | 1 |
|  | Tarn-et-Garonne | 1 |
| Experience |  |  |
|  | Less than 10 years | 3 |
|  | Between 10 and 30 years | 5 |
|  | More than 30 years | 7 |
| Modality of practice |  |  |
|  | Private | 9 GPs |
|  | Mixed private and public | 1 GP and 2 gynaecologists |
|  | Clinic/Hospital/Health centre | 2 gynaecologists |
|  | Multidisciplinary group practice/ Primary health care network | 6 midwives |
| Experience with women from deprived areas and/or non- French speaking women |  |  |
|  | Extensive (most patients) | 7 |
|  | Good (consequent part of the patients) | 7 |
|  | Fair (about 1/3 of patients) | 6 |
| Cervical cancer screening practice (GPs only) |  |  |
|  | Only prescription | 2 |
|  | Prescription and vaccination | 4 |
|  | Vaccination and screening | 4 |
| Use and recommendations of e-tools (apps, website) |  |  |
|  | Comfortable, regular recommendations to patients | 7 |
|  | Comfortable, occasional recommendations to patients | 3 |
|  | Comfortable, rare recommendations to patients | 5 |
|  | Non-user, no recommendation | 5 |

**Table S4:** Characteristics of participants in phase 2: women from deprived areas

| **Demographics** | **Categories** | **Number of participants**  **(total = 28)** |
| --- | --- | --- |
| Age |  |  |
|  | 30-44 years old | 12 |
|  | 45-54 years old | 5 |
|  | 55-65 years old | 11 |
| Family status |  |  |
|  | Single without children | 6 |
|  | Single with children | 8 |
|  | In a relationship without children | 1 |
|  | In a relationship with children | 9 |
|  | In a relationship with adult children | 4 |
| Area |  |  |
|  | Hérault | 14 |
|  | Gard | 5 |
|  | Pyrénées-Orientales | 4 |
|  | Haute-Garonne | 2 |
|  | Lot | 1 |
|  | Tarn | 1 |
|  | Tarn et Garonne | 1 |
| Professional status |  |  |
|  | Looking for employment | 5 |
|  | Unemployed/ housewife | 3 |
|  | Employed – part time | 8 |
|  | Employed – full time | 8 |
|  | Retired | 4 |
| Education level |  |  |
|  | Middle school | 3 |
|  | High school/ High school graduation | 14 |
|  | High school graduation + 1 or 2 years | 11 |
| Participation in cervical cancer screening |  |  |
|  | Never/ does not remember | 12 |
|  | More than 3 years ago | 12 |
|  | More than 10 years ago | 4 |

**Table S5:** Characteristics of participants in phase 2: healthcare professionals

| **Demographics** | **Categories** | **Number of participants**  **(total = 13)** |
| --- | --- | --- |
| Age |  |  |
|  | 30-44 years old | 6 |
|  | 45-54 years old | 4 |
|  | 55-65 years old | 3 |
| Speciality |  |  |
|  | General Practitioner (GP) | 6 (4 women/ 2 men) |
|  | Gynaecologist | 3 (2 women/ 1 man) |
|  | Midwife | 4 (3 women/ 1 man) |
| Area of practice |  |  |
|  | Hérault | 3 |
|  | Pyrénées-Orientales | 2 |
|  | Tarn | 2 |
|  | Aude | 1 |
|  | Aveyron | 1 |
|  | Haute-Garonne | 1 |
|  | Gard | 1 |
|  | Lozère | 1 |
|  | Tarn-et-Garonne | 1 |
| Experience |  |  |
|  | Less than 10 years | 5 |
|  | Between 10 and 25 years | 4 |
|  | More than 25 years | 4 |
| Modality of practice |  |  |
|  | Medical practice (shared or not) | 4 GPs |
|  | Private | 2 Gynaecologists |
|  | Mixed private and public | 1 Gynaecologist |
|  | Clinic/ Hospital | 1 GP and 1 midwife |
|  | Multidisciplinary group practice | 3 midwives |
| Experience with women from deprived areas and/or non- French speaking women |  |  |
|  | Extensive (most patients) | 2 |
|  | Good (consequent part of the patients) | 5 |
|  | Fair (about 1/3 of patients) | 6 |
| Cervical cancer screening practice (GPs only) |  |  |
|  | Only prescription | 2 |
|  | Prescription and vaccination | 4 |
| Use and recommendations of e-tools (apps, website) |  |  |
|  | Comfortable, regular recommendations to patients | 4 |
|  | Comfortable, occasional recommendations to patients | 2 |
|  | Comfortable, rare recommendations to patients | 3 |
|  | Non-user, no recommendation | 4 |

**Table S6:** Characteristics of participants in phase 3

| **Demographics** | **Categories** | **Number of participants**  **(total = 15)** |
| --- | --- | --- |
| Age |  |  |
|  | 30-44 years old | 5 |
|  | 45-54 years old | 5 |
|  | 55-65 years old | 5 |
| Family status |  |  |
|  | Single without children | 4 |
|  | Single with children | 1 |
|  | In a relationship without children | 3 |
|  | In a relationship with children | 3 |
|  | In a relationship with adult children | 4 |
| Area |  |  |
|  | Aveyron | 1 |
|  | Gard | 2 |
|  | Haute-Garonne | 3 |
|  | Hérault | 6 |
|  | Pyrénées-Orientales | 3 |
| Professional status |  |  |
|  | Looking for employment | 3 |
|  | Unemployed/ housewife | 1 |
|  | Employed – part time | 6 |
|  | Employed – full time | 4 |
|  | Retired | 1 |
| Education level |  |  |
|  | Middle school | 2 |
|  | High school/ High school graduation | 7 |
|  | High school graduation + 1 or 2 years | 6 |
| Participation in cervical cancer screening |  |  |
|  | Never/ does not remember | 6 |
|  | More than 5 years ago | 7 |
|  | More than 10 years ago | 2 |
